# Medial Hemiplateau High‐Tibial Osteotomy Technique for Varus Tibial Plateau Malunion

**DOI:** 10.1002/atn2.70329

**Published:** 2026-10-20

**Authors:** Imran Gruhonjic, Tim Wang

**Affiliations:** ^1^ Department of Orthopaedic Surgery, Sports Medicine Scripps Clinic Medical Group, Shiley Center for Orthopaedic Research and Education at Scripps Clinic La Jolla California U.S.A.

## Abstract

Tibial plateau fractures can be devastating injuries and lead to progressive pain, post‐traumatic arthritis, and joint instability. Although these are typically treated with open reduction internal fixation at the time of injury, occasionally persistent deformity and/or malunion can occur. In the older patient, knee arthroplasty can be an effective treatment, but in the young patient, joint preservation approaches should be attempted to allow return to desired levels of activities. The purpose of this technical note is to describe the preoperative planning and surgical technique in the treatment of complex post‐traumatic deformity using a 3‐dimensional‐printed patient‐specific guide and implant to correct medial tibial plateau varus malunion.

## Graphical abstract

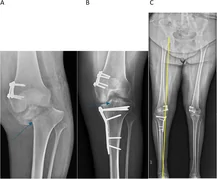

**VIDEO 1:** Medial high tibial hemiplateau osteotomy in the setting of varus malunion. Imaging of prior distal femoral‐guided growth for pre‐existing valgus deformity because of arthrofibrosis and metal artifact. Posterolateral corner injury with varus deformity shown on preoperative computed tomography (still image). A 3‐dimensional‐printed patient‐specific guide was placed on the anteromedial tibia using the tibial tubercle. A 5‐mm screw was drilled using a positive‐stop drill bit to fasten the guide to the tibia in the supine position. Preselected drill bit used through a guide to perforate the anterior cortex starting medially. Rivets on the anterior‐most aspect of the guide were removed; the superior portion was detached three‐fourths inch osteotome marked to the predetermined depth used to complete the osteotomy. Preoperative osteotomy appearance (still image), subchondral and parallel to the articular surface. Laminar spreader used to place Kirschner wires. Opening verified using a 3‐dimensional‐printed validator, and Kirschner wires drilled across to maintain alignment. Clinical and radiograph correction (still images). Plate positioned on medial tibia; fit confirmed using 3‐dimensional‐printed model. Cortical screw placed in plate guide to compress the plate to the tibial surface. Position of plate confirmed under fluoroscopy. Description of the remaining procedure with 2 partially threaded cannulator screws, allograft bone graft with final closure, final construct showing plate, and follow‐up radiograph (still images). Video content can be viewed at https://doi.org/10.1002/atn2.70329.

Varus and valgus deformities of the knee represent angular misalignments between the tibia and femur, leading to altered biomechanical loading across the joint. These malalignments disrupt the normal distribution of forces within the knee, contributing to progressive cartilage degeneration, osteoarthritis, and functional impairment. In patients with minimal cartilage wear, younger age, and suitability for joint‐preserving procedures, osteotomies have been employed to restore alignment. Whether performed at the distal femur or proximal tibia, these corrective procedures aim to redistribute mechanical loads, alleviate pain, and delay osteoarthritis progression.

For varus malalignment, high tibial osteotomy (HTO) remains the primary surgical approach.^1^, ^2^ Correction can be achieved via medial opening‐wedge or lateral closing‐wedge techniques, each with distinct advantages and limitations.^3^ The lateral closing‐wedge osteotomy involves resecting a bone wedge from the lateral proximal tibia, inherently enhancing stability and permitting earlier weight‐bearing.^4^ However, this technique may necessitate a fibular osteotomy and poses a greater risk of peroneal nerve injury.^5^ Conversely, the medial opening‐wedge osteotomy, which has gained increasing favor, involves creating a controlled osteotomy on the medial proximal tibia and progressively opening a wedge to achieve the desired correction. This approach mitigates complications associated with the lateral closing technique but is associated with increased risks of nonunion, tibial plateau fractures, and potential alterations in the posterior tibial slope.^6^, ^7^, ^8^

In an effort to enhance surgical precision and optimize outcomes, patient‐specific instrumentation (PSI) has been integrated into HTO procedures.^9^, ^10^ PSI employs preoperative imaging and computer‐assisted design to generate custom surgical guides tailored to an individual's anatomy. This technology enhances the accuracy of osteotomy cuts, ensures optimal alignment correction, and may reduce the operative time.^11^

The typical opening‐wedge HTO is performed from a medial approach, cutting 80% to 90% of the way to leave a lateral hinge to adjust the mechanical axis between the entire tibial plateau and the tibial shaft. In this technique guide, we describe a post‐traumatic varus deformity of the medial tibial plateau treated with medial opening‐wedge hemiplateau HTO using PSI.

## SURGICAL TECHNIQUE

Clinical examination, diagnostic imaging, and radiographic assessment should be obtained to comprehensively evaluate the patient's condition and history. Clinical examination of ligamentous structures and meniscus is critical to assess for concomitant soft‐tissue injury. Anteroposterior, lateral, and Rosenberg radiographs are used to visualize the joint space height. Long‐leg anteroposterior hip‐to‐ankle radiographs are necessary to determine the weight‐bearing axis and corresponding lateral distal femoral angle and medial proximal tibial angle (Figure 1). These measurements, in conjunction with the joint line congruence angle, will help determine the degree of correction for preoperative osteotomy planning. For PSI planning, computed tomography scan protocols are obtained to create further 3‐dimensional measurements of bony alignment and custom implant fabrication (Figure 2, Video 1).

**FIGURE 1:**
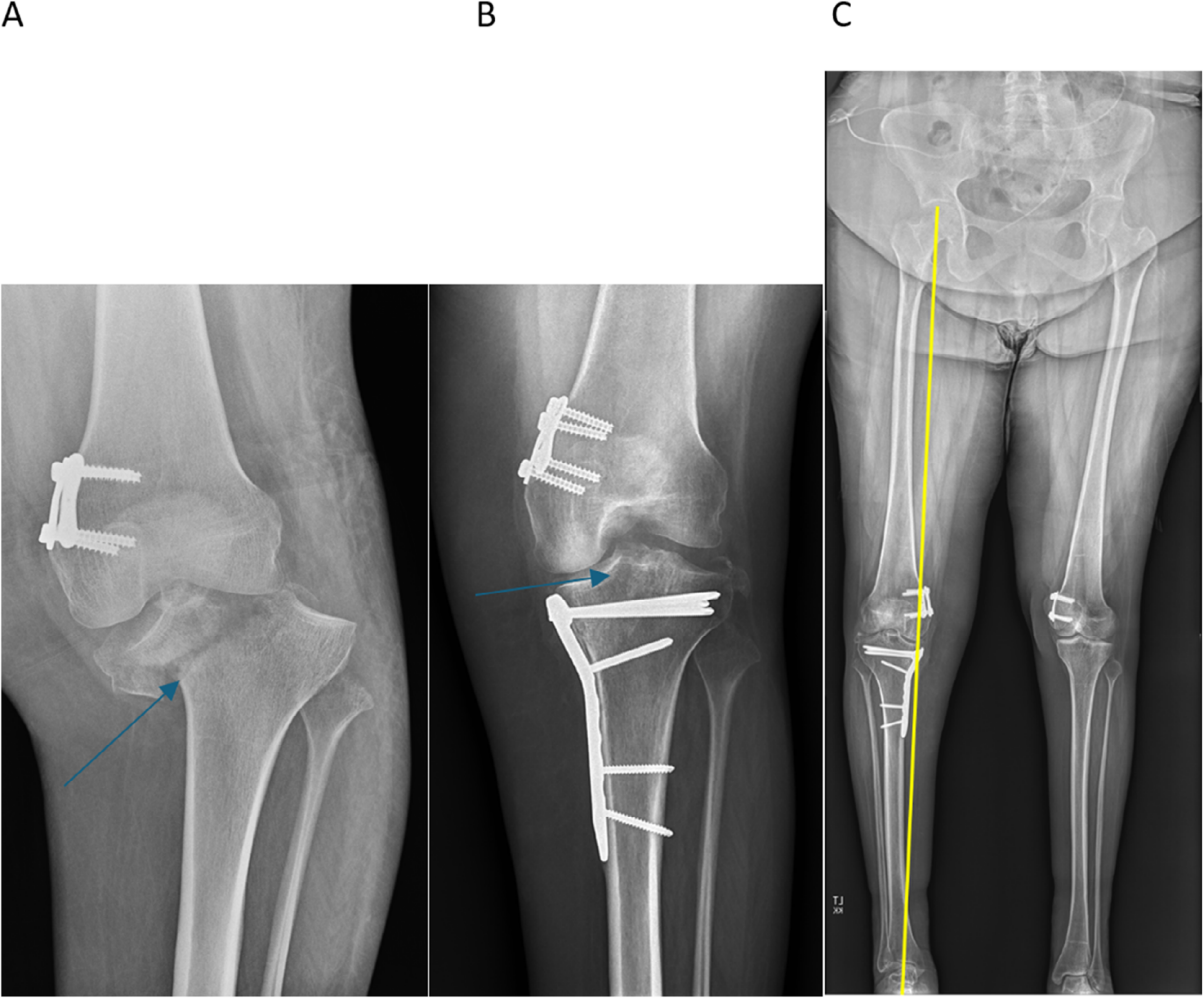
(A) Anteroposterior radiograph of the left knee at the time of the initial injury showing medial tibial plateau fracture. (B) Anteroposterior radiograph of the left knee 1 year postoperatively from facture medial tibial plateau open reduction internal fixation at outside facility showing joint line deformity and malunion. (C) Long‐leg anteroposterior hip‐to‐ankle radiograph showing previous femoral valgus deformity (lateral distal femoral angle), tibial varus deformity (medial proximal tibial angle), and overall varus mechanical axis fixation showing tibial medial plateau fracture and previously treated alignment (yellow line).

**FIGURE 2:**
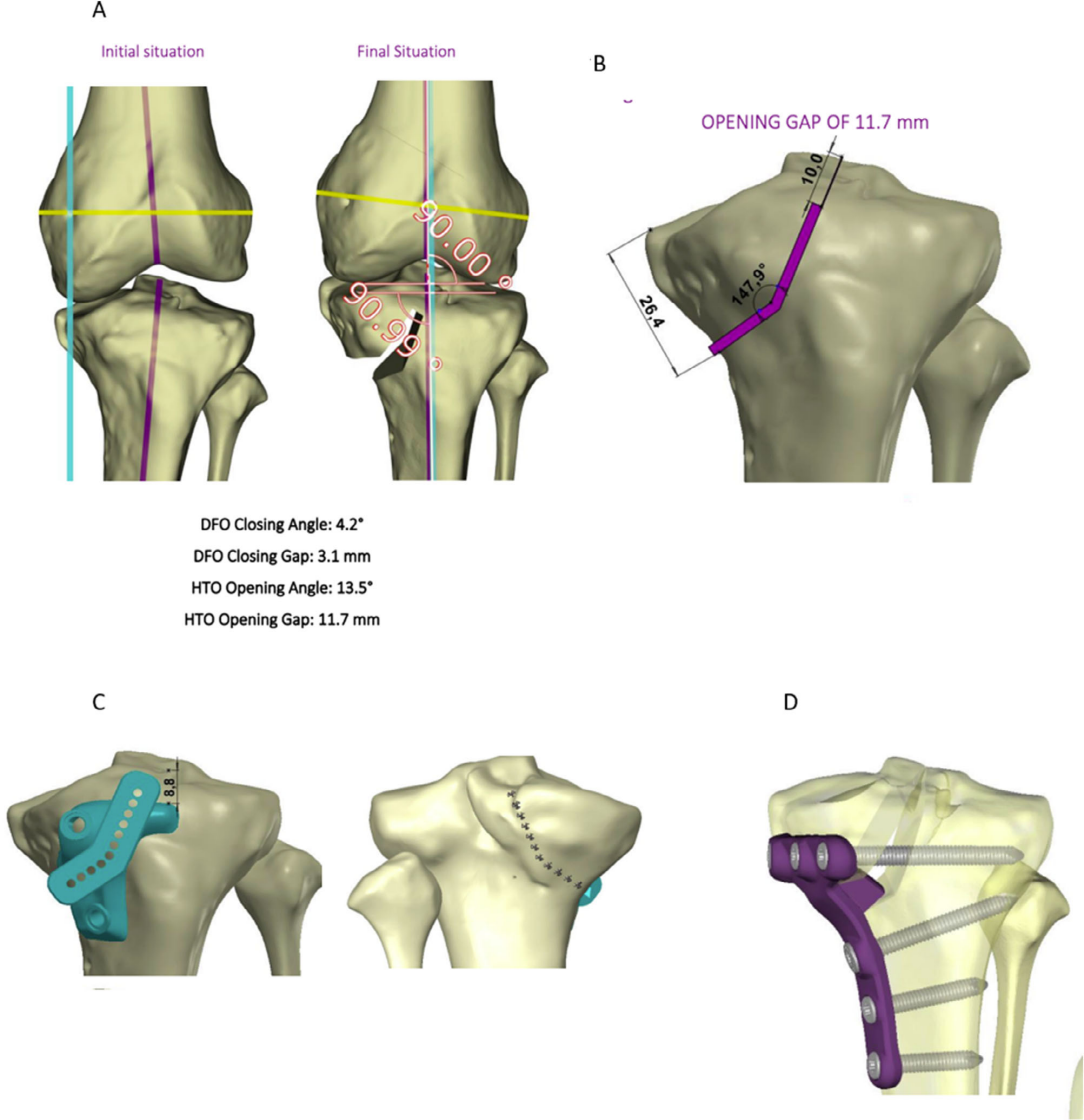
(A) Initial preoperative coronal CT‐based scan showing baseline anatomic measurements and planned postoperative correction. (B) Planned site of hemiplateau osteotomy as visualized on 3D‐based CT scan. (C) Planned location of custom cutting guide for osteotomy as visualized on 3D‐based CT scan. (D) Projected position of final plate and screw fixation with completed osteotomy. (3D, 3‐dimensional; CT, computed tomography; DFO, distal femoral osteotomy; HTO, high tibial osteotomy.)

### Anesthesia and Positioning

The patient is positioned in the supine position on the operating table and induced under general anesthesia. Single‐shot adductor canal blocks can be used for perioperative pain control. An examination under anesthesia is performed to validate the clinical examination findings. A well‐padded high‐thigh tourniquet is placed on the surgical leg. Perioperative intravenous antibiotics and tranexamic acid are administered for infection prophylaxis and hemostasis control, respectively (Tables 1 and 2).

**TABLE 1:** Potential Pearls and Pitfalls When Performing HTO Procedure With a Hemiplateau Technique Using Patient‐Specific Instrumentation

**Pearls**	**Pitfalls**
Careful preoperative planning to identify desired correction	Ensure the custom guide is positioned at the same location of preoperative plan using bony landmarks and radiographic appearance
Perform soft‐tissue dissection with the knee in flexion to allow for retraction of neurovascular structures	Careful attention to the articular surface so not to complete or displace the joint surface
Complete osteotomy with osteotomes to allow controlled cortical penetration	Slow, controlled opening of the osteotomy to prevent hinge fracture (1 mm per min)

HTO, high tibial osteotomy; min, minute.

**TABLE 2:** Advantages and Disadvantages of a PSI Hemiplateau Repair Technique When Compared With Traditional HTO Procedures

**Advantages**	**Disadvantages**
Increased accuracy of correction—within 0.3°	Need for preoperative CT scan
Decreased complications and neurovascular injury increased case efficiency	Additional cost for custom implant and planning
Enable increased case complexity	Timing for fabrication and planning
	Learning curve for surgeon

CT, computed tomography; HTO, high tibial osteotomy; PSI, patient‐specific instrumentation.

### Surgical Approach

Initial diagnostic arthroscopy is performed to assess intra‐articular structures and treat accordingly. An 8‐cm longitudinal incision is made along the anteromedial tibia from the joint line distally. The patellar tendon is carefully identified and protected throughout the exposure. An inverted L‐shaped periosteal and soft‐tissue flap is developed just distal to the articular margin and medial to the tibial tubercle, extending posteriorly to expose the posteromedial cortex of the proximal tibia. This is a full‐thickness flap that includes elevation of the superficial medial collateral ligament and pes anserine tendons. The soft tissue is meticulously elevated off the posterior tibial surface. Digital palpation is performed to confirm posterior retraction of the soft tissues of the posterior tibial surface and exposure to the fibular head and protected using a lap pad and a radiolucent Hohmann retractor (Figure 3). The knee is placed in flexion to allow the popliteal artery to retract posteriorly (Video 1).

**FIGURE 3:**
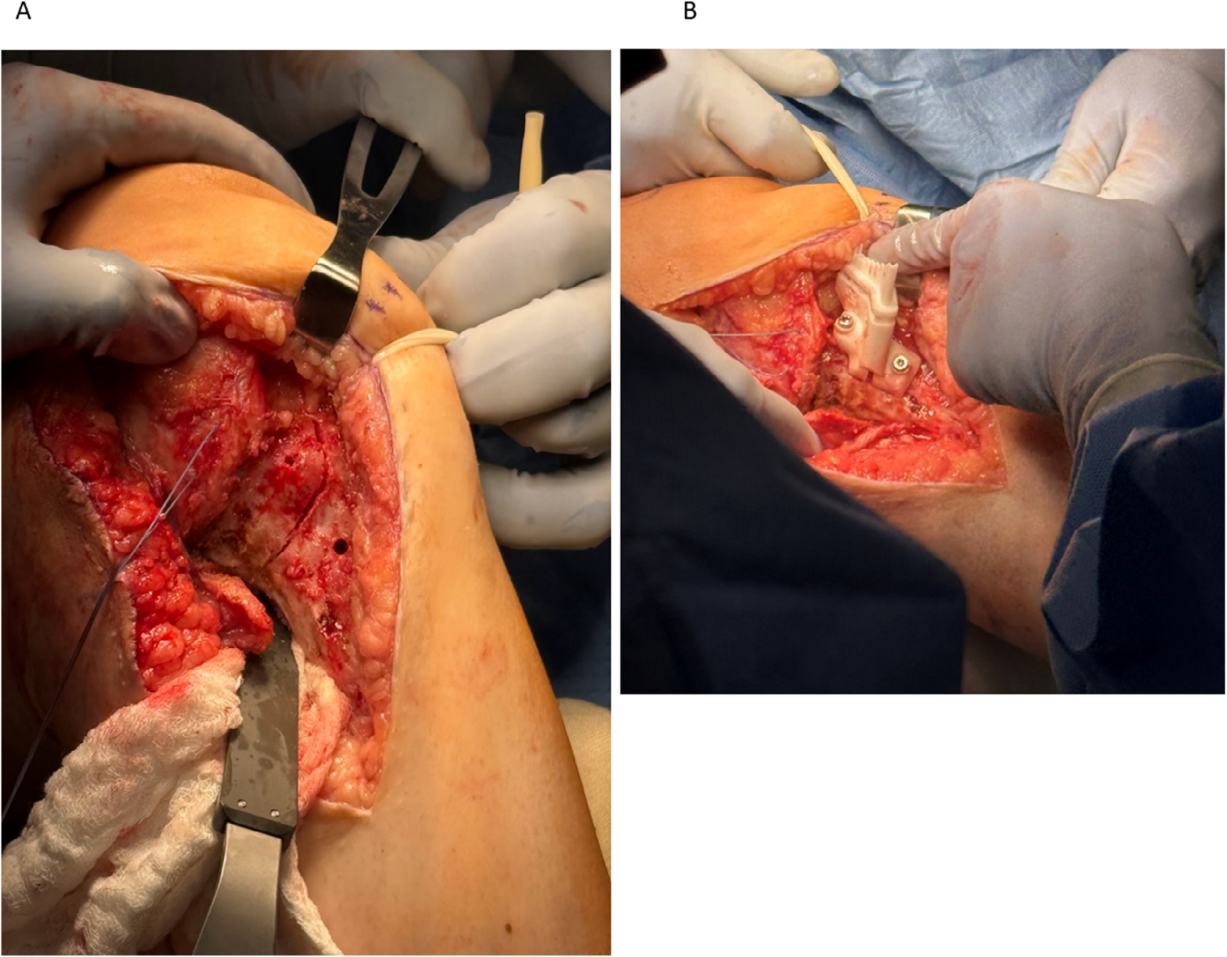
(A) Clinical photograph showing posterior soft‐tissue retraction and protection with radiolucent Hohmann retractor along the posteromedial tibia during medial hemiplateau tibial osteotomy. (B) Placement of custom drill guide on proximal tibia at preoperatively determined location in preparation for medial hemiplateau tibial osteotomy.

### Medial Tibial Osteotomy Technique

The custom PSI guide (created using the Bodycad Fine Osteotomy system using a standard computed tomography) is placed on the proximal medial tibia in the predesignated position, which was confirmed using palpation, a 3‐dimensional‐printed anatomical model, and correlation of Kirschner wire positioning with the preoperative plan (Figure 3). Using the precalibrated drill holes on the PSI guide, cortical perforations are made with a 2‐mm drill bit to define the osteotomy trajectory, and the osteotomy is completed under direct visualization using osteotomes (Video 1).

To avoid gapping or displacement at the articular surface, the osteotomy is carefully terminated just inferior to the articular margin (Figures 4 and 5). Two‐millimeter Kirschner wires are placed from the medial to the lateral, ending at the proximal hinge to preserve the integrity of the articular surface. The osteotomy site is gradually distracted using a laminar spreader until the predetermined correction was achieved (Figure 6). The custom‐designed locking plate with the metallic wedge is then positioned in accordance with the preoperative plan and secured with locking and nonlocking screws. Two 4.5‐mm cannulated screws are inserted parallel to the joint line at the joint level to compress and stabilize the articular surface (Figure 7). Allograft bone graft wedges are inserted into the osteotomy site to provide structural support and accelerate healing (Figure 8). The final correction is confirmed with the preoperative plan (Figure 9). If indicated, a concomitant medial closing‐wedge distal femoral osteotomy can be performed to treat pre‐existing valgus deformity of the femur (Video 1).

**FIGURE 4:**
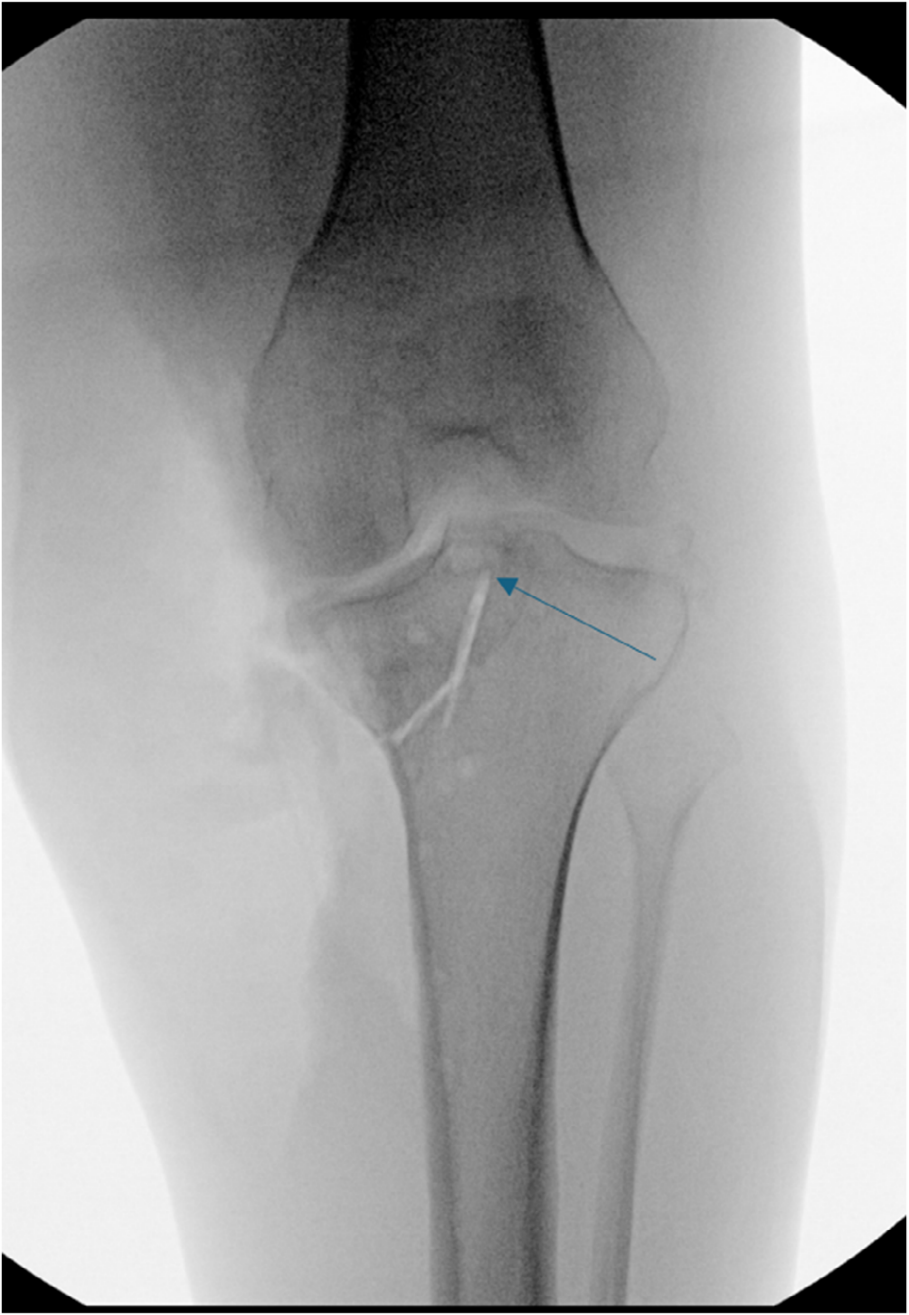
Intraoperative anteroposterior fluoroscopy image after medial hemiplateau tibial osteotomy is completed, just inferior to the joint line without penetration.

**FIGURE 5:**
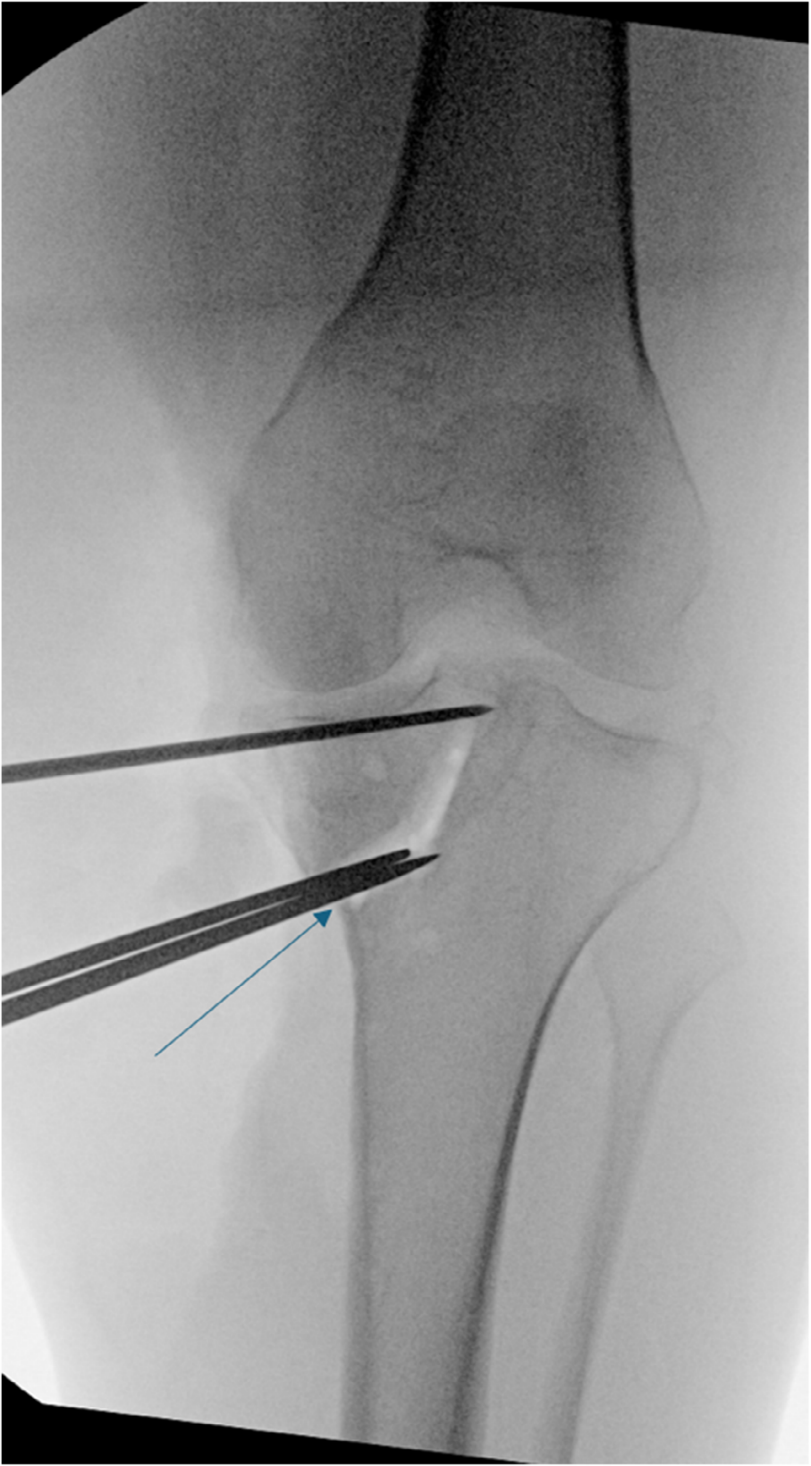
Anteroposterior intraoperative fluoroscopic image showing stacked osteotomes placed in the medial hemiplateau tibial osteotomy site to achieve appropriate restoration of alignment.

**FIGURE 6:**
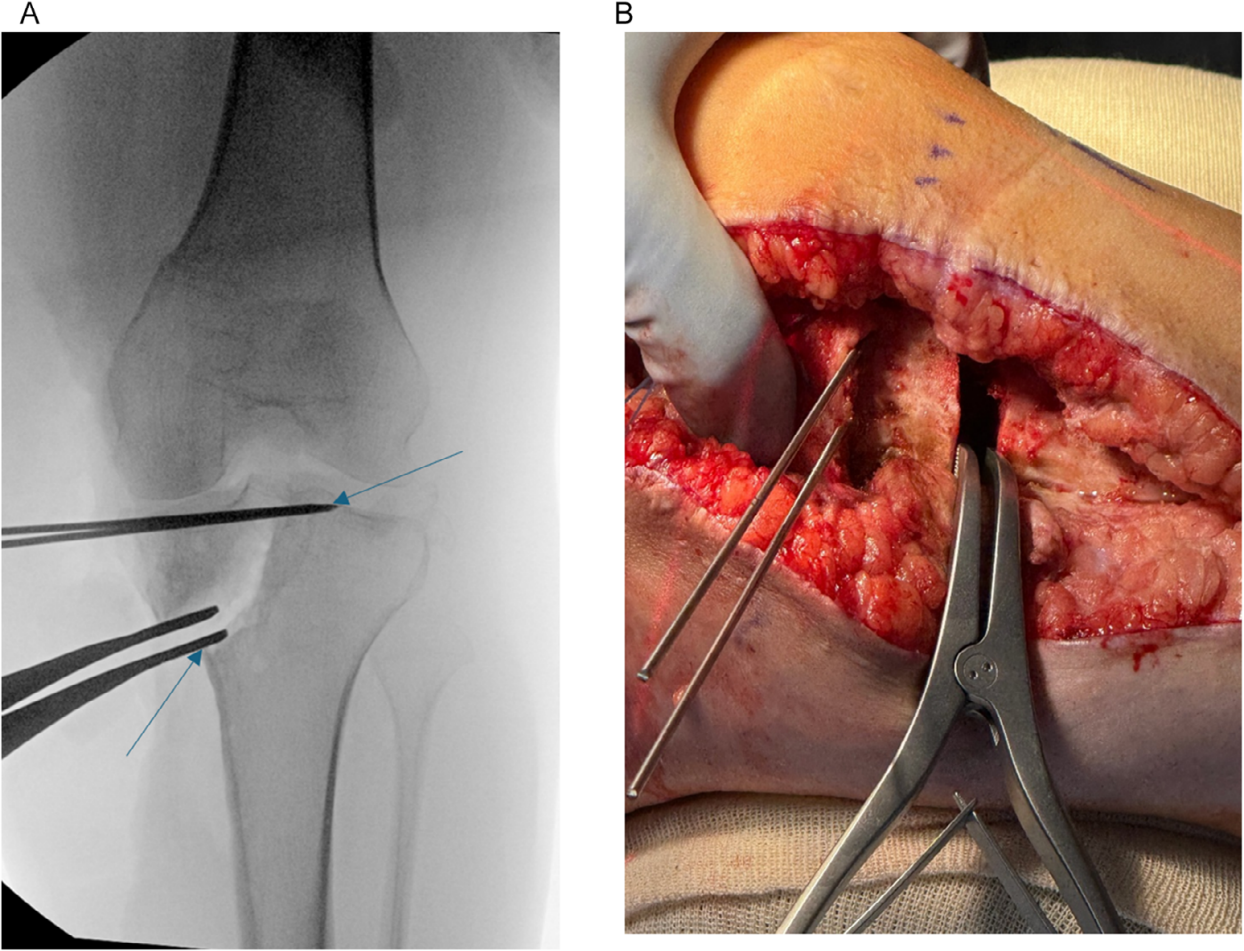
(A,B) Anteroposterior fluoroscopic image and clinical photograph viewing from the medial side of the tibia showing medial hemiplateau tibial osteotomy site gapping with a lamina spreader to the desired level of correction of alignment, with Kirschner wires placed parallel to the articular surface to prevent propagation into the joint.

**FIGURE 7:**
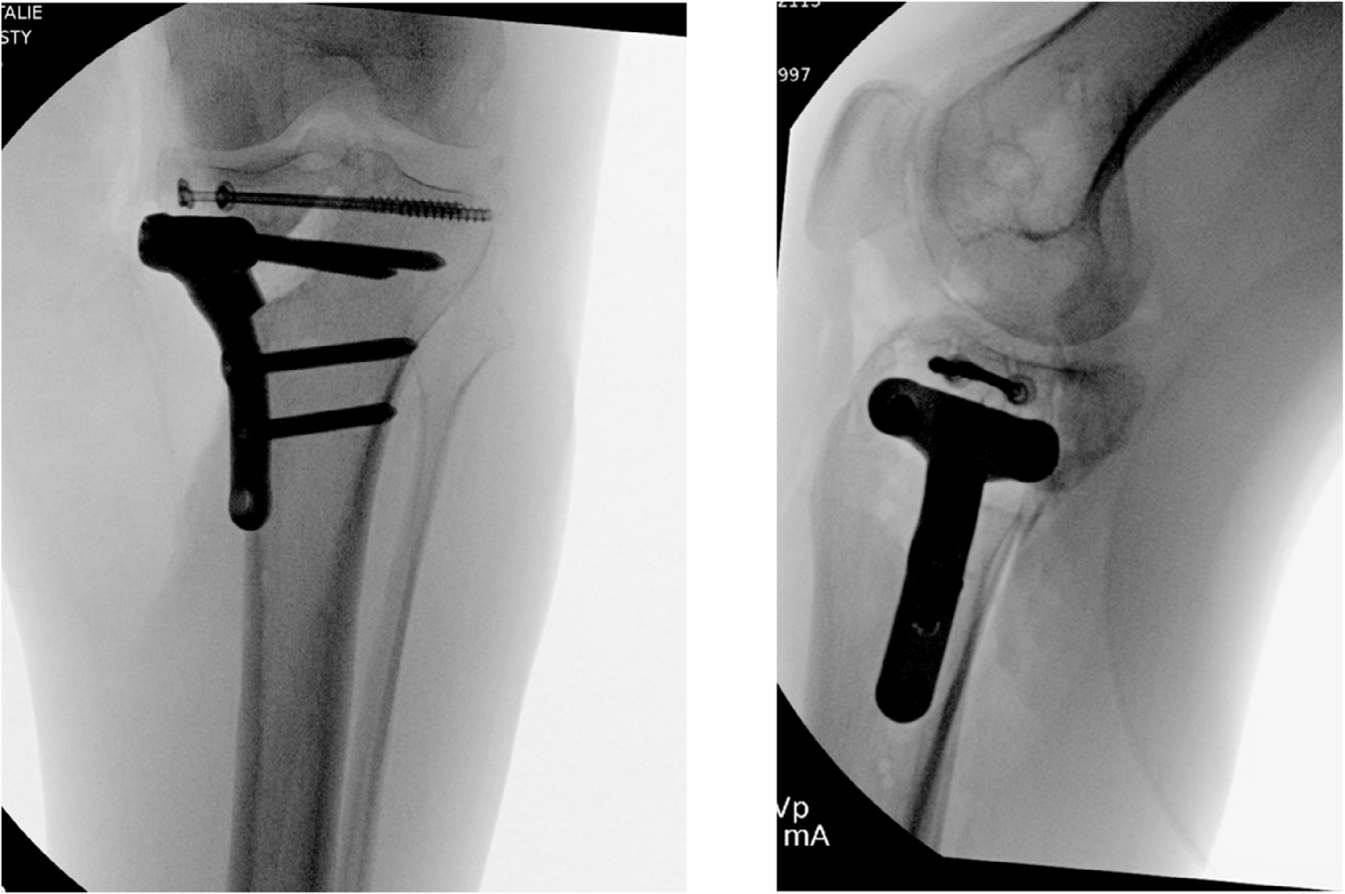
Anteroposterior and lateral intraoperative fluoroscopic views showing medial tibial plate fixation of hemiplateau osteotomy in addition to two 4.5‐mm cannulated screws inserted parallel to the joint line to compress and stabilize the articular surface.

**FIGURE 8:**
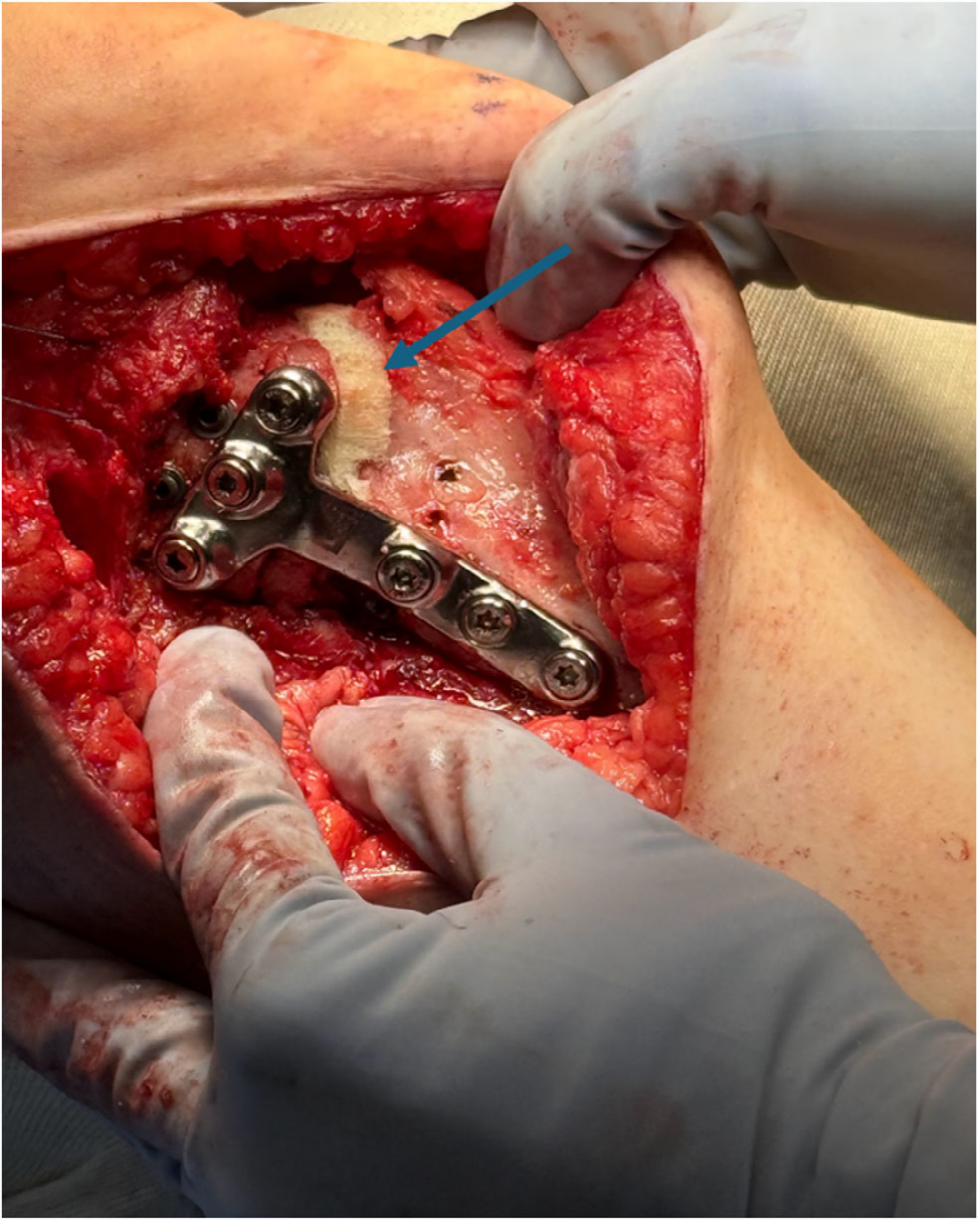
Clinical photograph viewing from the medial side showing allograft bone graft placement within the medial hemiplateau tibial osteotomy site with successful fixation/placement of medial tibial plate and screws.

**FIGURE 9:**
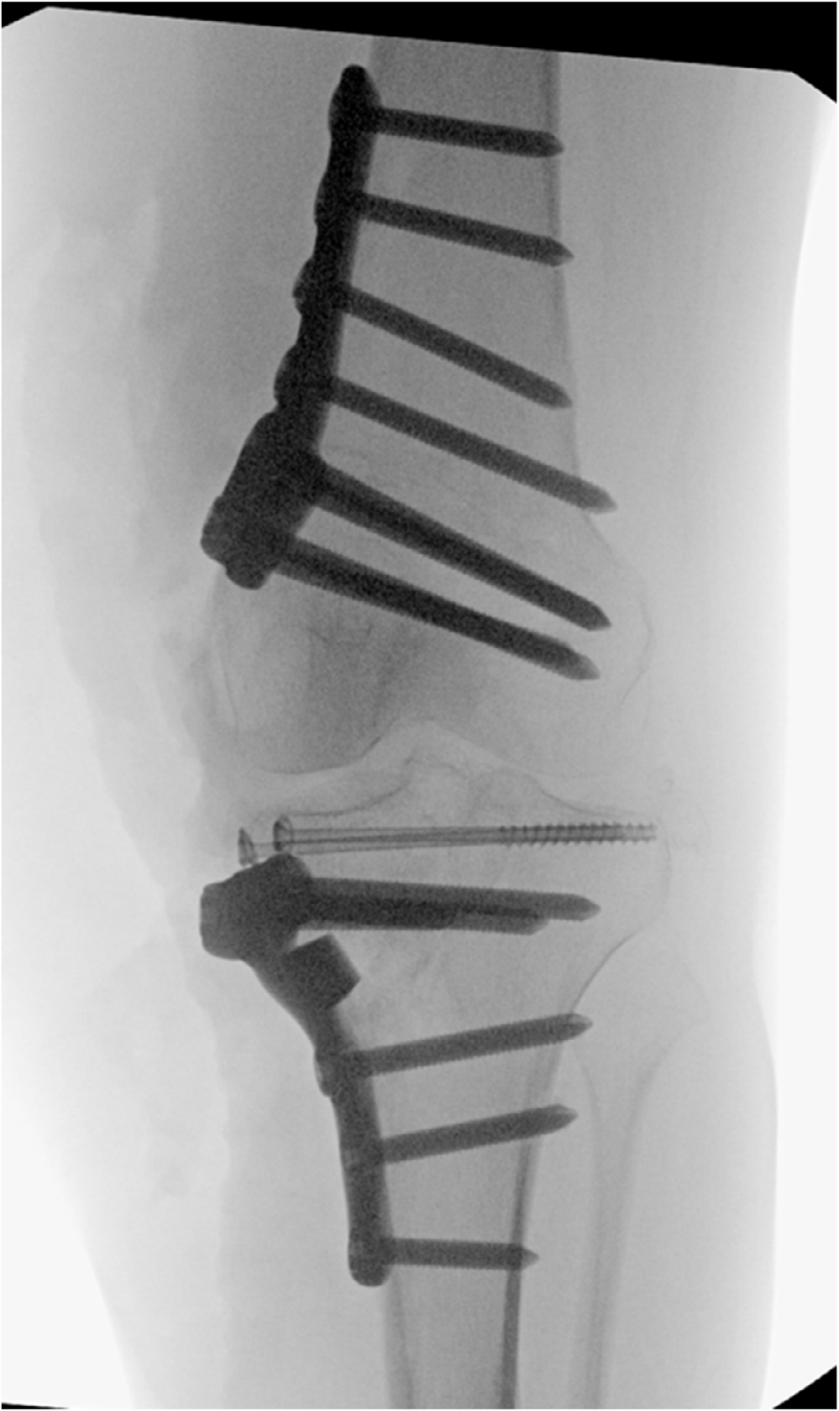
Anteroposterior intraoperative fluoroscopic image showing final construct of medial tibial hemiplateau opening‐wedge osteotomy with concomitant medial distal femoral closing‐wedge osteotomy to treat pre‐existing femoral valgus deformity.

### Closure

The tourniquet is let down, and hemostasis is achieved. The periosteal flap can be closed over the plate with side‐to‐side sutures or a small suture anchor to repair the pes anserine attachment. Superficial tissue and skin are closed per surgeon preference. The knee is placed in a hinged knee brace locked in full extension.

### Postoperative Rehabilitation

The patient is kept touchdown on the surgical extremity for 6 weeks. Radiographs are performed at 12 weeks postoperatively, and the patient is gradually progressing to partial and full weight‐bearing thereafter (Figure 10).

**FIGURE 10:**
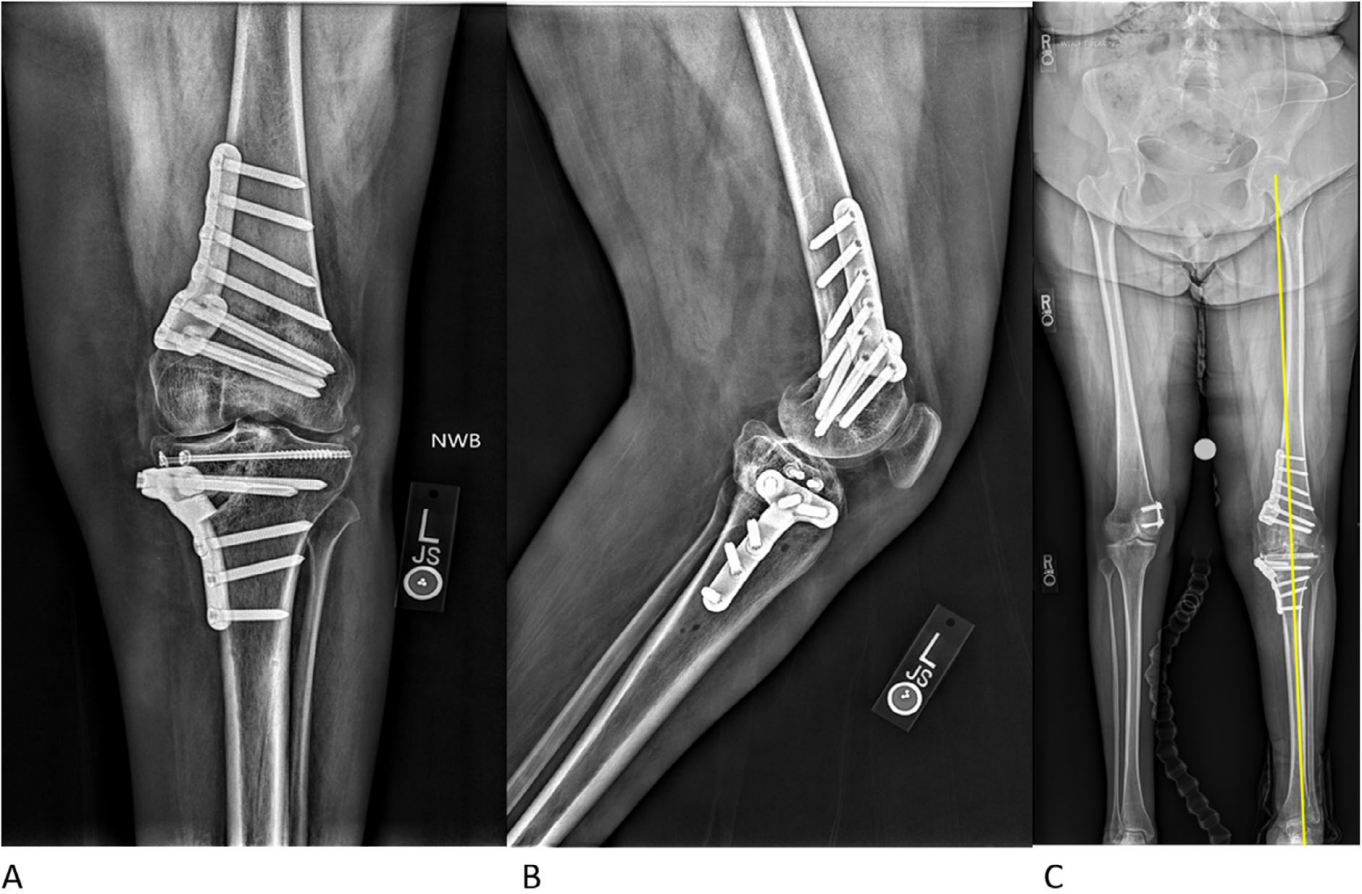
Three‐month postoperative anteroposterior (A), lateral (B), and long‐leg alignment (C) radiographs showing healed osteotomy sites and correction of deformity after medial hemiplateau opening‐wedge osteotomy and medial distal femoral closing‐wedge osteotomy.

## DISCUSSION

Malunion deformities of the knee can be profoundly debilitating because they disrupt the physiological distribution of mechanical forces across the joint. Such alterations in load distribution can accelerate cartilage degeneration, contribute to the development and progression of osteoarthritis, and lead to symptomatic instability and functional impairment. Corrective osteotomies performed through the proximal tibia, targeting both coronal and sagittal planes, have been shown to significantly improve joint biomechanics, thereby creating a more favorable environment for the preservation and function of cartilage, meniscal tissue, and ligamentous structures (Pullen).^12^

In this case, we present a case of a post‐traumatic medial plateau malunion. Treatment options in this situation include partial knee arthroplasty, hemiplateau osteochondral allograft (Schmidt),^13^ hemiplateau allograft (Alkhayarin),^14^ and extra‐articular HTO (Page).^15^

We have identified only a few briefly published studies reporting a hemiplateau HTO.^14^, ^16^, ^17^ As outlined in this article, the hemiplateau technique addresses the deformity through the recreation of the deformity to allow the most anatomic correction. This technique preserves more native bone and decreases the likelihood of lateral hinge fractures while still providing adequate deformity correction. Successful outcomes depend on meticulous preoperative planning and a comprehensive understanding of PSI utilization.

With continued advancements in surgical technology and technique, the precision of osteotomy planning and execution has markedly improved.^18^ A notable innovation in this domain is the introduction of PSI. We believe that PSI technology is incredibly beneficial in deformity surgery because it can allow (1) increased accuracy of correction, (2) decreased complications and risk of neurovascular injury, (3) increased surgical efficiency and reduced radiation exposure, and (4) increased case complexity. The downsides of this technology include the need for a preoperative CT scan, additional cost to manufacture custom implants, delay in scheduling because of implant fabrication, and a learning curve for surgeons. PSI technology in osteotomies includes the use of 3‐dimensional preoperative modeling and planning, patient‐specific cutting guides, and custom implants and screw trajectories.

HTOs are associated with a range of potential complications. According to Miltenberg et al.,^19^ medial‐based HTOs carry approximately 1 in 11 risks of lateral hinge fractures. Lateral‐based HTOs, in contrast, are associated with a higher risk of peroneal nerve injury. Additional complications, regardless of technique, can include infection, loss of correction, and nonunion. Reoperation rates have been reported to reach up to 15%, often because of symptomatic hardware necessitating removal.

## DISCLOSURES

The author (T.W.) declares the following financial interests/personal relationships which may be considered potential competing interests: T.W. reports consulting fees from Moximed and Arthrex; payment or honoraria for lectures, presentations, speaker's bureaus, manuscript writing, or educational events from Miach; stock or stock options from Overture Orthopedics; and other financial or nonfinancial interests from Miach. The other author (I.G.) declares that he has no known competing financial interests or personal relationships that could have appeared to influence the work reported in this article.
